# Microbiota in *Clostridioides difficile*-Associated Diarrhea: Comparison in Recurrent and Non-Recurrent Infections

**DOI:** 10.3390/biomedicines8090335

**Published:** 2020-09-08

**Authors:** Alessandra Gazzola, Simona Panelli, Marta Corbella, Cristina Merla, Francesco Comandatore, Annalisa De Silvestri, Antonio Piralla, Valentina Zuccaro, Claudio Bandi, Piero Marone, Patrizia Cambieri

**Affiliations:** 1 Infectious Diseases Unit, Fondazione IRCCS Policlinico San Matteo, 27100 Pavia, Italy; alessandra.gazzola@unimi.it (A.G.); v.zuccaro@smatteo.pv.it (V.Z.); 2 Department of Veterinary Medicine, University of Milano, 20133 Milan, Italy; 3 Department of Biomedical and Clinical Sciences “L. Sacco” and Pediatric Clinical Research Center “Romeo ed Enrica Invernizzi”, University of Milano, 20157 Milan, Italy; francesco.comandatore@unimi.it; 4 Microbiology and Virology Unit, Fondazione IRCCS Policlinico San Matteo, 27100 Pavia, Italy; M.Corbella@smatteo.pv.it (M.C.); c.merla@smatteo.pv.it (C.M.); a.piralla@smatteo.pv.it (A.P.); p.marone@smatteo.pv.it (P.M.); p.cambieri@smatteo.pv.it (P.C.); 5 Clinical Epidemiology and Biometry Unit, Fondazione IRCCS Policlinico san Matteo, 27100 Pavia, Italy; A.DeSilvestri@smatteo.pv.it; 6 Department of Biosciences and Pediatric Clinical Research Center “Romeo ed Enrica Invernizzi”, University of Milano, 20157 Milan, Italy; claudio.bandi@unimi.it

**Keywords:** microbiota, *Clostridioides difficile*, recurrent *Clostridioides difficile* infection, diarrhea, antibiotics

## Abstract

*Clostridioides difficile* infection (CDI) is the leading cause of antibiotic-associated diarrhea, especially in hospitalized elderly patients, representing a global public health concern. Clinical presentations vary from mild diarrhea to severe pseudomembranous colitis that may progress to toxic megacolon or intestinal perforation. Antibiotic therapy is recognized as a risk factor and exacerbates dysbiosis of the intestinal microbiota, whose role in CDI is increasingly acknowledged. A clinically challenging complication is the development of recurrent disease (rCDI). In this study, using amplicon metagenomics, we compared the fecal microbiota of CDI and rCDI patients (sampled at initial and recurrent episode) and of non-infected controls. We also investigated whether CDI severity relates to specific microbiota compositions. rCDI patients showed a significantly decreased bacterial diversity as compared to controls (*p* < 0.01). The taxonomic composition presented significant shifts: both CDI and rCDI patients displayed significantly increased frequencies of *Firmicutes*, *Peptostreptococcaceae*, *Clostridium XI*, *Clostridium XVIII*, and *Enterococcaceae*. *Porphyromonadaceae* and, within it, *Parabacteroides* displayed opposite behaviors in CDI and rCDI, appearing discriminant between the two. Finally, the second episode of rCDI was characterized by significant shifts of unclassified *Clostridiales*, *Escherichia/Shigella* and *Veillonella*. No peculiar taxa composition correlated with the severity of infection, likely reflecting the role of host-related factors in determining severity.

## 1. Introduction

*Clostridioides difficile* infection (CDI) is the leading cause of infectious diarrhea, especially in hospitalized patients and in the community setting, representing a significant health problem worldwide. CDI is associated with a wide range of clinical features, from asymptomatic colonization to mild diarrhea and to the more severe pseudomembranous colitis that may progress to toxic megacolon, intestinal perforation, sepsis, and death [1]. Although most hospitalized patients infected by *C. difficile* are asymptomatic carriers, this population serves as a reservoir for continued *C. difficile* contamination of the hospital environment [2]. Indeed, the contact with spore-contaminated surfaces is one route by which this pathogen spreads to new hosts. *C. difficile* spores are transformed into an active state within the gastrointestinal tract once ingested [3]. During gut colonization, *C. difficile* releases the toxins that damage the enteric mucosal lining, by inducing apoptosis and necrosis of epithelial cells, contributing substantially to the inflammatory picture [4].

Recognized risk factors are numerous and include age (>65 years), underlying diseases, gastrointestinal surgery, long-term care residency, previous hospitalization, nasogastric tube feeding and use of drugs like antibiotics, proton-pump inhibitors (PPI) and histamine receptors 2 blockers, [1]. CDI is also often observed as a complication of severe comorbidities, especially intestinal inflammatory conditions as inflammatory bowel disease (IBD), where the association of *C. difficile* toxins with flares has been suggested [5].

The association of antibiotics with CDI has been firmly established in both the hospital and community settings [6,7]. Nearly all classes of antibiotics have been associated with CDI, but clindamycin, penicillins, cephalosporins, and fluoroquinolones seem to pose the greatest risk, followed by macrolides and sulphonamides/trimethoprim [1,7].

Standard treatment for *C. difficile*-associated diarrhea (CDAD) involves the administration of metronidazole or vancomycin. Although most cases respond initially to either drug, recurrence (rCDI) can occur after the discontinuation of therapy. As many as 25% of patients treated for an initial infection experience a recurrent infection within 30 days, and the risk can double after two or more recurrences [3]. rCDI is defined as a relapse of CDI symptoms within two to eight weeks of successful treatment of the initial episode. Recurrences are clinically challenging, and may result in repeated hospitalizations, malnourishment, and fecal incontinence. Although multiple factors are associated with recurrences, the exact contribution of each remains largely undetermined [8]. Antimicrobial treatment of rCDI yields success rates between 30% and 80%, depending on the number of recurrences, administered antibiotic and treatment duration [9]. Currently, fecal microbiota transplantation is the reference for CDI recurrences [10].

The prolonged use of antibiotics often administered to infected patients creates the relevant side effect of maintaining and exacerbating a dysbiotic state of the intestinal microbiota [11]. Indeed, the important role exerted by resident gastrointestinal bacterial communities in the development of CDI has been largely addressed in the last few years, and specific changes in bacterial compositions are believed to contribute to the susceptibility to CDI and rCDI [8]. It is now known that, normally, the indigenous gut microflora is refractory to colonization by external “invasive” species through a process known as “colonization resistance” [12]. Instead, the development of CDAD is thought to represent the breakdown of such colonization resistance, in which antibiotics play a major role, with their ability to induce profound and long-lasting changes in microbiota composition [2,11]. These changes include reduced biodiversity, loss of specific taxa, and shifts in metabolic capacities and last from six months to two years after treatment [2,13]. The decreased diversity mainly affects the dominant *Bacteroidetes* and *Firmicutes* phyla and is in turn accompanied by an overgrowth of *Proteobacteria*. Concerning taxonomic changes, the loss of specific combinations and ratios between specific taxa plays a role in favoring the persistence of *C. difficile*, rather than the presence or absence of single species [2]. For example, several species within the *Eubacterium* genus, together with some *Clostridium* species, notably *Clostridium scindens*, inhibit *C. difficile* infection thanks to their metabolic activities. The altered equilibrium among these species strongly favors the persistence of CDI [1]. Finally, disruption in microbiota composition is profound in patients with rCDI who have received multiple antibiotic courses [14].

The present study aimed to characterize the fecal microbiota of patients infected by *C. difficile*. Subjects with non-recurrent (CDI) and recurrent (rCDI) infection were considered and compared to non-infected controls. Both initial (rCDI1) and secondary (rCDI2) episodes of diarrhea were considered for patients with recurrent infection. The threat of rCDI is one of the most increasingly common challenges in the management of CDI and predicting which patients are at a higher risk of recurrences is clinically crucial. For this reason, we verified if specific features of the gut microbiota within our cohort characterized rCDI.

Another crucial point is to establish if differential microbiota signatures may be associated with the severity of the infection. Taxonomic composition and diversity of the gut microbiota in all infected patients (CDI and rCDI) were thus related to the severity of the infection, expressed according to the Zar score [15]. The use of scoring systems for categorizing disease severity is common in clinics and is based on laboratory and clinical variables (e.g., age of the patient, temperature, hematological values, presence of complications such as pseudomembranous colitis or the need for treatment in the intensive care unit.). It guides therapeutic choices and is used for predicting response to therapy and clinical outcomes. For these important practical implications, and because the Zar score is a widely used scoring system, we used it to group *C. difficile* infections as “severe” and “non severe”, in order to evaluate possible correlations with peculiar compositions or properties of the gut microbiota. This is an original point of the present work and is potentially relevant for the clinical practice.

## 2. Results

A total of 40 subjects were enrolled. They included 10 cases of single *C. difficile* infection (CDI) and the same number of recurrent infection (rCDI), for which samples collected at both first (rCDI1) and second (rCDI2) episode of diarrhea were available. Twenty subjects, tested for being non-infected by *C. difficile*, age- and sex-matched, served as controls (C). The demographic and clinical features of all cases are shown in Table 1, and antibiotic therapies are detailed in Table 2. Zar scores calculated for both CDI and rCDI patients are listed in Table 2.

### 2.1. Taxonomic Structure of Fecal Bacterial Communities in CDI and rCDI Patients Compared to Non-Infected Controls

In order to investigate shifts in structure and composition of fecal bacterial communities across patient groups and compare CDI and rCDI cases, 50 amplicons (20 from non-infected controls, C, 10 from CDI, 10 for rCDI1, and 10 rCDI2), comprising the V1–V3 regions of 16S rRNA gene, underwent sequencing and processing. The demographic and pathological features of the enrolled subjects are reported in Materials and Methods (see below). A total of 3.9 million reads were obtained and clustered into 2391 OTUs at 97% homology level. After application of low count and low variance filters, OTUs were representative of 12 bacterial phyla, 23 classes, 35 orders, 71 families, and 139 genera. Supplementary Tables S1–S4 report the relative abundances of, respectively, phyla, classes, orders, families and genera in individual samples.

**Figure 1:**
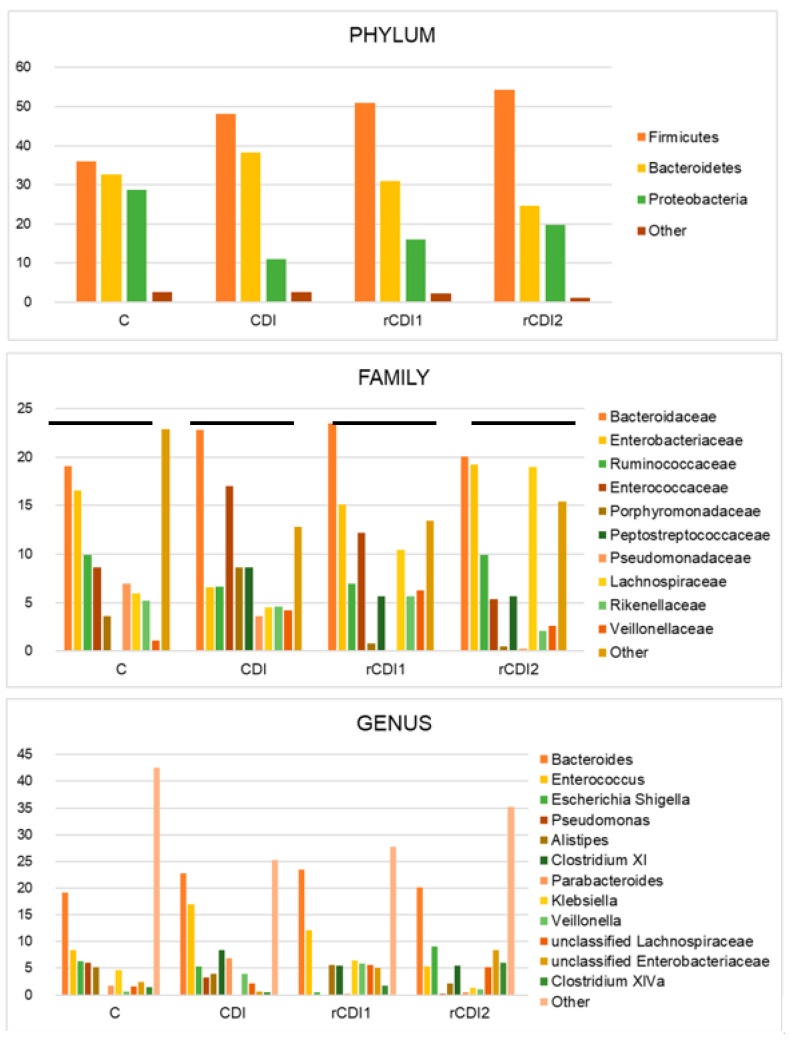
Taxonomic composition of the gut microbiota in non-recurrent and recurrent *C. difficile* infection as compared to non-infected controls. Average relative abundances of the most represented phyla, families and genera identified in the four study groups. C = non-infected controls; CDI = non-recurrent *C. difficile* infection; rCDI1 = recurrent *C. difficile* infection, first episode; rCDI2 = recurrent *C. difficile* infection, second episode. Only taxa whose relative abundance was >5% in at least one group were included.

The average relative abundance for the most represented phyla, families and genera is shown in Figure 1. As for phyla, *Firmicutes* and *Bacteroidetes* are the most abundant and, together with *Proteobacteria*, account for nearly all the bacterial diversity. In C, these three phyla present abundances around 30% (35.9% *Firmicutes*; 32.7% *Bacteroidetes*; 28.7% *Proteobacteria*). In CDI, both *Firmicutes* (48.1%) and *Bacteroidetes* (38.2%) frequencies are higher than in C. Patients suffering from refractory infection show a further expansion of *Firmicutes*: (50.9% and 54.3% at respectively the first and the second episode). *Proteobacteria* also increase as compared to CDI (16% in rCDI1 and 19.8% in rCDI2 vs. 11% in CDI). Finally, it is worth signaling the reduction of *Bacteroidetes* that characterize rCDI (30.9% and 24.7% respectively at first and second episode of infection, vs. 38.2% in CDI patients). Concerning the taxonomic rank of families, the most abundant in C are *Bacteroidaceae* (19.1%) and *Enterobacteriaceae* (16.6%), followed by *Ruminococcaceae* (9.9%), *Enterococcaceae* (8.7%), *Pseudomonadaceae* (6.9%), *Lachnospiraceae* (5.9%), and *Rikenellaceae* (5.2%). The infection with *C. difficile* is accompanied by an expansion of *Enterococcaceae* (17% vs. 8.7% in C, 12.2% and 5.3% respectively in rCDI1 and rCDI2) and *Porphyromonadaceae*, (8.7% vs. 3.7% in C, and values < 1% in both rCDI1 and rCDI2). The *Clostridiales* family of *Peptostreptococcaceae* reaches 8.6% in CDI and 5.6% in both rCDI1 and rCDI2. Lastly, rCDI is characterized by an increase in *Lachnospiraceae* (order: *Clostridiales*), as compared to controls (10.4% and 19% respectively in rCDI1 and rCDI2 vs. 5.9% in C). For refractory patients, it should also be noted the expansion of *Enterobacteriaceae* (15.1% in rCDI1 and 19.3% in rCDI2) as compared to CDI (6.6%). With regards to genera, *Bacteroides* is the most abundant in all groups, with an increasing trend in patients infected by *C. difficile* (prevalences: 19.1% in C; 22.8% in CDI; 23.5% in rCDI1; 20.1% in rCDI2). *Enterococcus* increases as well in CDI (16.9%) and rCDI1 (12.2%) as compared to controls (8.3%). *Parabacteroides* is detected in CDI with a prevalence of 6.9%, whereas it is nearly undetectable in the other groups. *Clostridium XI*, the *Peptostreptococcaceae* lineage comprehending *C. difficile*, is detected only in infected patients (8.4% in CDI, and 5.5% for both rCDI1 and rCDI2). The recurrent infection is marked by an increased *Escherichia/Shigella* (9% in rCDI2 vs. 5.4% in CDI and 6.3% in C). *Klebsiella* increases in rCDI1 (6.5%) as compared to rCDI2 (1.3%) and CDI (0.1%) and is detected in C (4.7%) as well. *Veillonella* reaches its maximum abundance in rCDI1 (5.9%) and is detected in CDI and rCDI2 as well (4% and 1.1%, respectively). Lastly, *Clostridium XIVa* is mainly represented in the recurrent diarrhea episode of refractory patients, rCDI2 (6.1%, vs. 1.4% in C; 0.5% in CDI; 1.7% in rCDI1).

**Figure 2:**
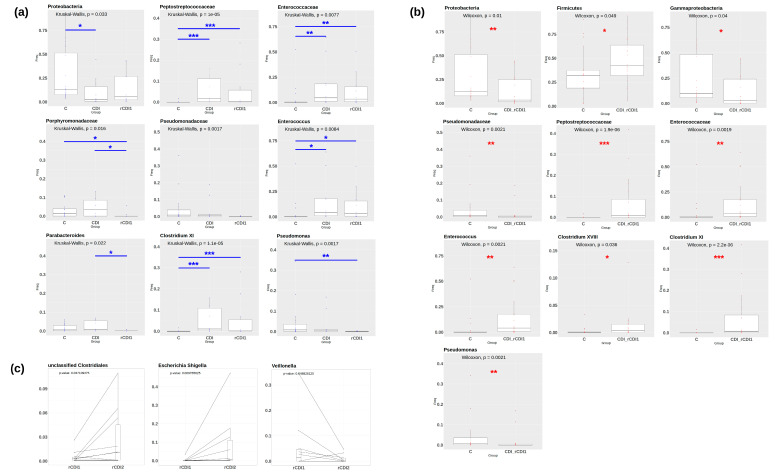
Taxa displaying significantly different relative abundances in the following comparisons: C vs. CDI vs. rCDI1 (**a**); C vs. CDI + rCDI1 (**b**); rCDI1 vs. rCDI2 (**c**). Pairwise comparisons producing significant *p*-values are indicated as * (*p* < 0.05); ** (*p* < 0.01); *** (*p* < 0.001). The cut-off value for taxa prevalence was set at 10%. Comparisons between patient categories are indicated over the boxplots. Values for paired Wilcoxon (C) are indicated in red. C = non-infected controls; CDI = non-recurrent *C. difficile* infection; rCDI1 = recurrent *C. difficile* infection, first episode; rCDI2 = recurrent *C. difficile* infection, second episode.

To better define differential taxa abundance, evidencing statistically significant differences in bacterial composition among controls, non-recurrent and recurrent patients, Mann–Whitney U-test and Kruskal–Wallis Rank Sum non-parametric tests were applied, using a 10% cut-off for prevalence. The following classes of patients were compared: (i) C vs. CDI vs. rCDI1 to highlight differences between non recurrent and recurrent infections, besides comparing them to controls (Figure 2A), (ii) C vs. CDI + rCDI1, to gain a comprehensive view of taxonomic changes associated with the infected state (Figure 2B). Finally, the two episodes of diarrhea for each rCDI patient (i.e., rCDI1 and rCDI2) were compared by Wilcoxon signed-rank test (Figure 2C, *p*-values are reported on the graphs). For all statistical analyses, significance threshold (*p*-value) was set to 0.05. When more than two groups are compared the obtained *p*-values were corrected using the Benjamini–Hochberg *post hoc* test.

From Figure 2A, it is evident that the first episode of recurrent CDI is characterized by a statistically significant decrease in the *Porphyromonadaceae* family and, within this family, of the genus *Parabacteroides* as compared to non-recurrent CDI. On the contrary, the infected state (CDI + rCDI1) is characterized by a strong increase in *Firmicutes* and, in particular, of the *Peptostreptococcaceae* family (Figure 2B). *Clostridium XI* (the phylogenetic lineage comprising *C. difficile*, belonging to *Peptostreptococcaceae*), *Clostridium XVIII, Enterococcaceae*, and *Enterococcus* are significantly increased as well. On the contrary, *Pseudomonadaceae* and *Pseudomonas* are significantly reduced. Finally, the second episode of recurrent CDI, when compared to the first one, is accompanied by a significant increase in both unclassified *Clostridiales* and *Escherichia/Shigella*, and by a decrease in *Veillonella* (Figure 2C). Other, less abundant, taxa displaying significantly differential abundances in comparisons (i) and (ii) (see above) are shown in Supplementary Figure S1.

### 2.2. Ecological Analyses of Fecal Communities in Infected and Non-Infected Patients

**Figure 3:**
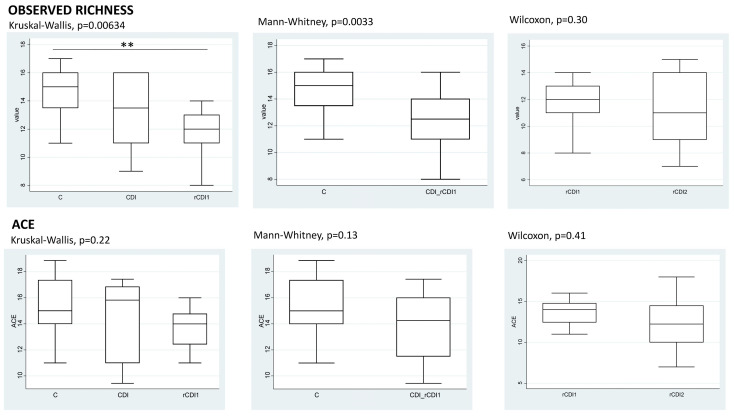

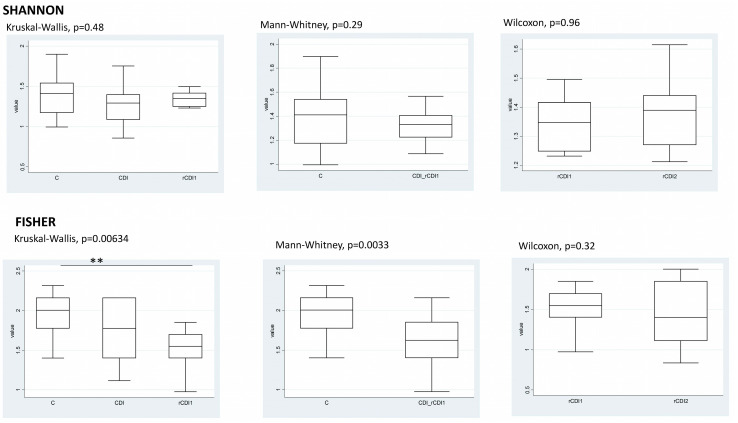
α-diversity. Overall comparison of microbiota structure. Observed richness, abundance coverage estimator (ACE), Shannon and Fisher indices are presented at the taxonomic level of classes. Significant (** *p* < 0.01) comparisons between patient categories are indicated over the boxplots. Abbreviations for patient cohorts: C = non-infected controls; CDI = non-recurrent *C. difficile* infection; rCDI1 = recurrent *C. difficile* infection, first episode; rCDI2 = recurrent *C. difficile* infection, second episode.

The within-sample diversity (α-diversity) was evaluated by computing observed richness, ACE, Shannon and Fisher indices. Figure 3 reports the boxplots for the taxonomic rank of classes and for the following comparisons: (i) C vs. CDI vs. rCDI1; (ii) C vs. CDI + rCDI1; (iii) rCDI1 vs. rCDI2 using the Wilcoxon rank-sum test for paired data. The purposes of these comparisons and the clinical questions linked to each of them have been explained in Section 2.1.

While a decreasing trend can be observed in both CDI and rCDI, a critical reduction and statistical significance (*p* < 0.01) are reached only for the latter, considering C vs. rCDI comparisons and both observed richness and Fisher indexes.

**Figure 4:**
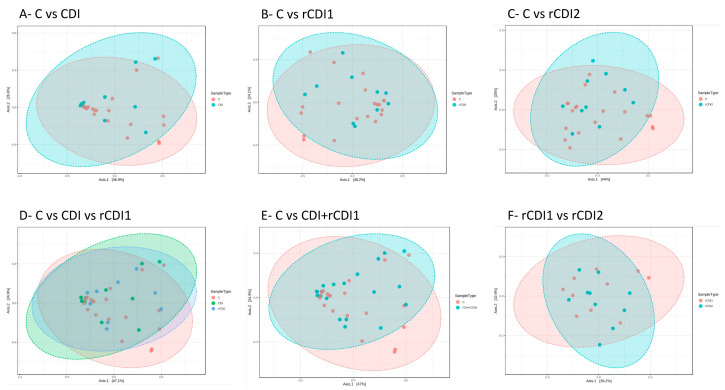
β-diversity. The microbiota distances were evaluated through the Bray–Curtis dissimilarity matrix at the taxonomic level of classes and visualized through Principal Coordinates Analysis (PCoA). Each point represents the microbiota composition of one sample. Abbreviations for patient cohorts: C = non-infected controls; CDI = non-recurrent *C. difficile* infection; rCDI1 = recurrent *C. difficile* infection, first episode; rCDI2 = recurrent *C. difficile* infection, second episode.

The between-sample diversity (β-diversity) was evaluated by computing the Bray–Curtis dissimilarity matrix and visualized through PCoA, both considering the three comparisons already used for α-diversity analysis and following pairwise comparisons: C vs. CDI, C vs. rCDI, CDI vs. rCDI. The analyses suggested a similar overall structure for all bacterial consortia, without a clear clustering of patient groups classes. This was observed for taxonomic rank of classes (Figure 4) as well as for all other ranks.

### 2.3. Taxonomic Composition and Diversity of the Gut Microbiota in Relation to the Severity of C. difficile Infection

We then tested if significant differences in composition of bacterial taxa and/or in ecological indexes of diversity could be retrieved if infections were categorized on the basis of their severity, as is routinely performed in the current clinical practice with prognostic purposes and to guide therapeutic choices. Thus, based on the widely used Zar score [15] (see Section 4), the 30 episodes of *C. difficile* infection included in the present study, comprehending 10 CDI and 10 rCDI (considered at both initial and recurrent episodes) were retrospectively clustered as severe (S) and non-severe (NS) (see Section 4).

At initial episode of infection, considering both CDI and rCDI1 groups, 9 out of 20 (45%) patients had an S disease (Zar score ≥ 2), and 11 (55%) an NS infection (Zar score < 2). Among the 9 S patients, 6 (66.7%) experienced an rCDI, whereas among the 11 NS, there were 4 rCDI (36.4%).

**Figure 5:**
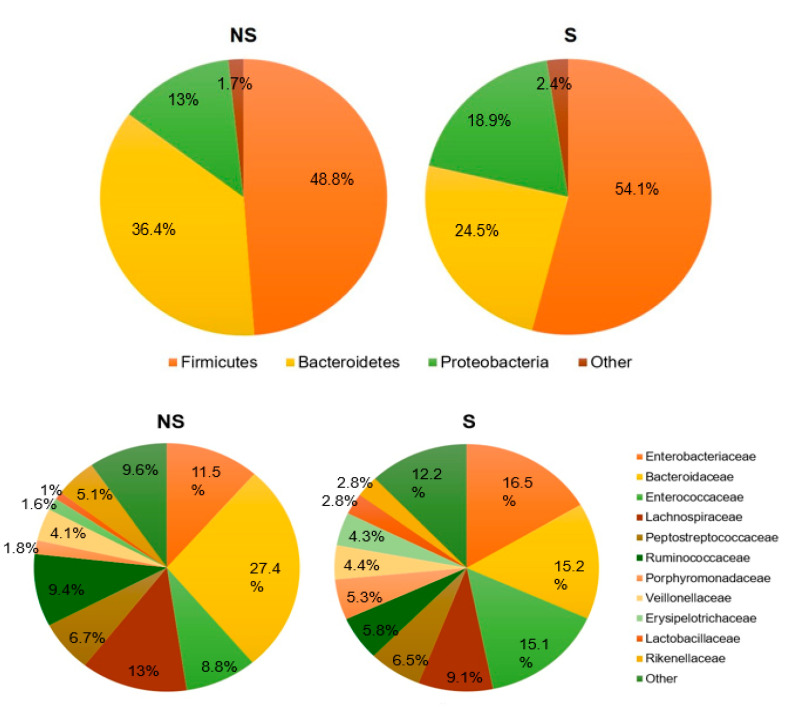

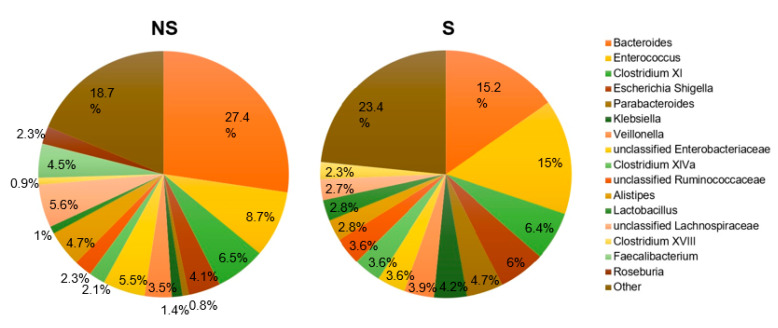
Comparison of taxonomic composition of microbiota in severe and non-severe *C. difficile* infection. Compared average relative abundances of phyla, families and genera identified in the two study groups. NS = non severe disease; S = severe disease.

Figure 5 shows pie charts for the two categories, considering the taxonomic levels of phyla, families and genera. Statistical analyses (conducted as described above) revealed that no significant differences were observed in S and NS infections either in microbiota taxonomic composition or in ecological indexes of α- and β-diversity when clustering patients based on disease severity.

## 3. Discussion

Infection by *C. difficile* is a global and increasingly common public health problem and is considered the major cause of antibiotic-associated diarrhea, especially in healthcare settings and in elderly individuals over the age of 65. CDI has known dramatic increases in severity and incidence over the past decade, with frequent outbreaks, especially in hospitals, reported both in Europe and in the United States [5]. Of growing concern is also the increasing incidence of CDI in categories not traditionally considered “at risk”, as children and young adults. It has been calculated that approximately 40% of patients acquiring community-associated CDI have no prior antibiotic exposure [16]. While the pathogenesis of *C. difficile* is not entirely clear, it is well recognized that functional alterations in the gastrointestinal microbiota contribute to the creation of a favorable environment for *C. difficile* colonization. The persistence of a dysbiotic state following antibiotic therapies or continued assumptions of other drugs such as PPI, constitutes a risk factor and frequently leads to the development of recurrences of infection. [11,16,17].

In the present work, we aimed to define changes in microbiota ecological properties and taxonomic composition associated with *C. difficile* infection, and to compare patients suffering from non-recurrent and recurrent infection to assess if specific microbial signatures could predict treatment response and recurrence.

Concerning the ecological properties of bacterial communities, our results indicate that *C. difficile* infection is accompanied by a decrease in biodiversity indexes compared to non-infected controls, with a maximum (and statistical significance) in recurrent patients, in accordance with previous results [10,11,16,18].

With regards to taxonomic composition, our data show that infection by *C. difficile* is accompanied by significant alterations in the abundance of some taxa, mainly belonging to *Firmicutes*, generally confirming previous findings of metagenomics works on CDI and contributing to the increase of records [5,10,11,16,18,19,20]. In our patients, the infected state considered globally (CDI and rCDI1) share most of the significant shifts in microbiota structure, probably also due to the size of the cohorts. The infected state is associated with significant increases of *Peptostreptococcaceae* and *Clostridium incertae sedis XI*, which is the phylogenetic cluster containing *C. difficile.* The enrichment in *Clostridium cluster XI* in CDI has been linked to the fact that, besides containing *C. difficile*, this cluster includes some opportunistic species [18]. An interesting result from our data is the significant increase of *Clostridium incertae sedis XVIII* in all infected patients. The effects of *Clostridium XVIII* are controversial, as some species produce exotoxins and have a pro-inflammatory potential, while other act as probiotics [21]. Increase of *Clostridium XVIII* is reported in various pro-inflammatory and pathological conditions, as in gastric cancer patients in the perioperative period [22] or in those with high fat diets [23]. Its increase in CDI has been sporadically reported [20], but this taxon is not commonly listed as associated with CDI. Our findings highlight the need to further investigate its role in *C. difficile* infection, while defining which specific members of this cluster increase in abundance. Outside the *Clostridium* universe, other taxa increased upon infection (CDI and rCDI1) are *Enterococcaceae* and *Enterococcus,* as already reported e.g., [5,8,10,18,19].

It is worth noting that some taxa display opposite trends in CDI and rCDI1 cohorts, despite the limited number of patients. *Porphyromonadaceae* (phylum: *Bacteroidetes*) and, within it, the genus *Parabacteroides* constitute an example. In our cohorts, the gut commensal *Parabacteroides* strongly decreases in rCDI1 as compared to CDI, where instead it presents the maximum abundance. Interestingly, previous studies have reported significantly higher rates of *Parabacteroides* in patients who responded to therapy as compared to non-responders, but the role of this genus in *C. difficile* infection, especially in recurrent forms, remains controversial [20]. Our findings disagree with a previous report [24], suggesting the increase of *Parabacteroides* as a predictor of recurrence. The discrepancy could be due to specific characteristics of cohorts (clinics, therapy, demographics, etc.), especially in the control group, and to the relatively high taxonomic ranks normally considered when performing amplicon metagenomics.

A further goal of our work was to assess if different microbiota compositions could be retrieved when analyzing the first and the recurrent diarrhea episode in rCDI patients. Most interestingly all patients sampled at the recurrent diarrhea showed an expansion in *Enterobacteriaceae* (especially in the pathobionts *Escherichia/Shigella*) from the first to the second episode. Our results support the clinically relevant finding that this taxon may play a role in predicting recurrences, as suggested previously [24]. Its increase during CDI has been linked with clindamycin treatment [8,24]. A taxon displaying the opposite pattern (i.e., a significant decrease passing from rCDI1 to rCDI2) is *Veillonella* (family: *Veillonellaceae*). In this regard, it seems reasonable to link this finding to published data pointing to a strong positive correlation between *Veillonella* spp. and *C. difficile* abundances [25]. Indeed, as shown by a recent in vitro study, *Veillonella* increases when a dysbiotic microbiota is co-cultivated with *C. difficile* [26]. However, it remains unclear whether *Veillonella* has a direct role in CDI development, e.g., via biofilm formation, or whether it simply increases as a result of altered metabolic pathways and/or unoccupied niches in the gut due to antibiotic use or *C. difficile* expansion [24,27]. An increase in *Veillonella*, even if not statistically significant, is also observed when comparing the non-refractory vs. refractory conditions (i.e., CDI vs. rCDI). Overall, these observations support the idea that this taxon could be considered predictor of recurrence, as previously suggested by Khanna and colleagues (2016) [24].

A final goal was to establish if differential microbiota features were associated with different severities of the disease, expressed by means of scoring systems. These are widely used in clinical practice for categorizing the severity of *C. difficile* infections, assisting in therapeutic choices, and predicting responses to therapy and clinical outcomes [28]. Our results suggest that the severity of a *C. difficile* infection, calculated based on the widely used Zar score, does not correlate either with a peculiar microbiota composition, or with a significant reduction in its diversity. Instead, several other factors could account for disease severity, in primis host-related factors, such as genetic variants involved in the regulation of the immune landscape (either at the levels of innate or adaptive immunity), or in epithelial barrier function [29,30]. For example, the regulation of host humoral immunity plays a key role in disease progression and outcome [29]: individuals with a greater antibody response to CDI toxins are more likely to be asymptomatic carriers, while those with lower antibody levels will more likely develop diarrhea and a severe disease after initial colonization. The innate immune response has been hypothesized to play a role as well, as *C. difficile* exerts its pathogenicity primarily through enterotoxins against which the host mounts an inflammatory response through different pathways that can influence the severity of CDI [31]. Severity and outcome of CDI could finally be influenced by communities of gut inhabitants (or transient passengers) not addressed in the present study, such as gut fungi. Recently, it has been shown that oral *Candida albicans* administration in a *C. difficile* mouse model worsens disease severity, exacerbating several markers used to characterize the severity of CDI (i.e., mortality rate, weight loss, gut leakage, and serum and intestinal tissue cytokines), without increased fecal *C. difficile* or bacteremia detection [32].

In summary, the identification of gut microbiota signatures may certainly be useful to predict recurrence in CDI and to define patients who need a more effective treatment to mitigate this risk. In the present work, we identified fecal microbiota changes associated with *C. difficile* infection, in part shared and in part differential between CDI and rCDI patients. On the contrary, microbiota data were substantially overlapping when categorizing patients based on the severity of infection. Even though the microbial community dynamics behind recurrences may be specific to the individual subject, further studies on larger cohorts are needed to understand the interactions of specific taxa with *C. difficile*. This interplay may be a key factor in determining the outcome of treatment, thus helping the physician to intervene more effectively.

## 4. Materials and Methods

### 4.1. Study Population

This study was a retrospective cohort study of patients with CDI, both with single *C. difficile* infection (CDI) and recurrent infection (rCDI), and subject tested for being non-infected by *C. difficile*, age- and sex-matched, and chosen among those available at the microbiology laboratory which served as controls (C). In the case of recurrent infection, samples were collected both at first (rCDI1) and second episode (rCDI2). The period of recruitment ranged from December 2015 to March 2019.

Diagnoses of CDI and rCDI were made based on widely accepted criteria which include the presence of diarrhea and a positive result of a laboratory test, as recommended by the Infectious Diseases Society of America (IDSA) [33]. These criteria, applied to each patient, resulted in the categorization of infections into non-recurrences (stated as CDI) and recurrences (subsequent positive sample 15–56 days from a previous positive sample) or reinfections (subsequent positive sample >56 days from a previous positive sample). To support a diagnosis of CDI or rCDI, a multistep algorithm exploiting the detection of glutamate dehydrogenase (GDH), the detection of toxins (A and B) and nucleic acid amplification test (NAAT), was used.

Infected patients were retrospectively stratified based on the severity of *C. difficile* infection, using the score developed in the clinical trial conducted by Zar et al. 2017 [15], the so-called Zar score. This severity score is attributed as follows: one point each is assigned for age > 60 years, temperature > 38.8 °C, albumin level < 2.5 mg/dL or peripheral White Blood Cell count > 15,000 cells/mm^3^. Two points are given for endoscopic evidence of pseudomembranous colitis or treatment in the intensive care unit. Patients with ≥2 points are considered to have severe CDI. Clinical data were extracted from the electronic medical record through both automated query and manual chart review by clinicians. The Zar scores calculated for both CDI and rCDI patients are listed in Table 2.

The protocol was approved by the local Ethics Committee (protocol number 20200026805).

### 4.2. Biological Samples

Stool samples from patients with confirmed diagnosis of CDI and rCDI (2 samples for each patient, collected at first and second episode), as well as those of controls, were kept at −80 °C, at the Laboratory of Microbiology and Virology of IRCCS Foundation “Policlinico San Matteo” of Pavia, and had been previously investigated for the presence of other pathogens during diagnostic routine.

### 4.3. Extraction of DNA and Production of 16S rRNA Amplicons (V1–V3 Regions)

DNA was extracted from fecal samples using the commercial QIAamp^®^ Fast DNA Stool Mini kit purchased from Qiagen (Hilden, DE, USA) and the protocol suggested by the manufacturer. The DNA concentration of each sample was assessed using a Qubit 3 fluorometer (Invitrogen, Carlsbad, CA, USA). The V1–V3 hypervariable regions of the prokaryotic 16S rRNA gene were targeted for amplicon production and sequencing was conducted by Arrows Diagnostics (Genova, Italy) using a paired end, 2 × 250-bp cycle run on an Illumina MiSeq sequencing system.

### 4.4. Bioinformatics and Statistical Analysis

Raw reads were processed using an ad-hoc, proprietary, bioinformatics pipeline (Arrow Diagnostics) built under the R environment and the Microbiome Analyst v. 3.5.1 (www.microbiomeanalyst.ca) online tool. Sequences were quality-filtered and classified into Operational Taxonomic Units (OTUs) at 97% homology level. Taxonomy was then assigned against the Ribosomal Database Project (RDP) reference database, release 11. Low-count (20% prevalence cut-off) and low-variance (based on the inter-quartile range) filters were applied, and data rarefaction and scaling (through total sum normalization) was performed, again using the default parameters of Microbiome Analyst. Filtered OTUs were used to compute relative abundances of microbial taxa in each sample. Microbial profiles of taxa with prevalence >10% in the dataset were compared among patient groups using the Mann-Whitney U-test and/or the Kruskal-Wallis Rank Sum test. In further detail: (a) comparison among C, CDI and rCDI groups was performed using Kruskal-Wallis Rank Sum test and Mann-Whitney U-test with Benjamini-Hochberg *post hoc* correction; (b) comparison between C and CDI_rCDI1 (where CDI and rCDI1 samples are grouped) was performed using Mann-Whitney U-test; (c) rCDI1 and rCDI2 were compared using Wilcoxon signed ranks test. For all statistical analyses, significance threshold (*p*-value) was set to 0.05.

The following α-diversity indexes were computed at all taxonomic ranks to analyze the within-sample diversity: observed richness, abundance coverage estimator (ACE), Shannon, Fisher. The obtained values were compared among selected groups using Kruskal-Wallis Rank Sum test and Mann-Whitney U-test with a significance threshold (*p*-value) set to 0.05% and Benjamini-Hochberg *post hoc* correction.

Diversity in composition among samples (β-diversity) was computed at all taxonomic ranks using the Bray-Curtis distance method and visualized as principal coordinates analysis (PCoA) on the resulting dissimilarity matrices. Permutational Multivariate Analysis of Variance (PERMANOVA) was performed for β–diversity analysis to assess the grouping of samples.

Limited to infected patients, microbial profiles and α-diversity values were also compared for two classes (not-severe, severe) obtained following the application of the Zar score to stratify patients (see above). Multilevel (episode and patient) population averaged generalized equation models were fitted to take into account the clustered nature of the data (autocorrelation of first order).

## Figures and Tables

**Table 1:** Demographic and clinical features of study cohorts. Abbreviations: CDI = patients suffering from non-recurrent *C. difficile* infection; rCDI = patients suffering from recurrent *C. difficile* infection; C = non-infected controls.

	Infected Patients			Controls
	CDI (10 Cases)	rCDI (10 Cases)		C (20 Cases)
Number of Cases	10	10		20
Male: Female Ratio	6:4	6:4		7:13
Mean Age in Years (+/− standard deviation)	67 +/− 18	66 +/− 16		66 +/− 17
Co-Morbidities:				
No Comorbidities°	1	0		7
Digestive	2	2		6
Cardio-Vascular	7	10		8
Respiratory	1	4		4
Endocrine-Metabolic	2	6		8
Neoplasia	1	1		3
Other	5	8	9	8
Drugs Other than Antibiotics:		Ep 1	Ep 2	
Protonic Pump Inhibitors	8	9	8	12/15
Nonsteroidal Anti-Inflammatory Drugs	0	0	1	1/14
Enteral Nutrition	0/9	2	2	0/12
Antibiotics in the Last 6 Months	6/8	7/10	10/10	6/15
Penicillins	3	5		4
Cephalosporins/Carbapenems	3	1		
Fluoroquinolones	2	2		3
Aminoglycosides				
Glycopeptides	1			1
Tetracyclines	1			
Macrolides	1	2		
Metronidazole				2
Others				4
NA	5	3		13
Antibiotics Other than Antibiotics for Treating *C. difficile*	7/10	5/10		8/14
Penicillins	4	2		3
Cephalosporins/Carbapenems	5	3		1
Fluoroquinolones	3			1
Aminoglycosides	1	1		
Glycopeptides	1			1
Tetracyclines				
Macrolides				1
Metronidazole				1
Others	1	1		2
NA	3	6		13
Previous hospitalization (6 months)	8/10	7/10	10/10	5/15

C vs. CDI vs. rCDI *p* = 0.05, C vs. rCDI *p* = 0.033.

**Table 2:** Characteristics and treatment of *Clostridioides difficile* infection in non-refractory and refractory patients. Abbreviations: CDI = patients suffering from non-recurrent *C. difficile* infection; rCDI1 = patients suffering from recurrent *C. difficile* infection, first episode; rCDI2 = patients suffering from recurrent *C. difficile* infection, second episode.

	CDI (Cases 10)	rCDI1 (Cases 10)	rCDI2 (Cases 10)
Type of Infection:			
Nosocomial ^1^	5	5	3
Community Associated ^2^	5	5	7
Antibiotics for Treating *C. difficile* (duration in days)			
Metronidazole	1 (9)	0	0
Vancomycin	6 (10)	8 (12)	7 (13)
Vancomycin + Metronidazole	1 (7)	1 (NA)	0
Fidaxomicin	0	1 (9)	0
Not known	2 (NA)	1 (NA)	3 (NA)
Zar-score:			
0–1	7	4	6
≥2	3	6	4

^1^ Symptoms appear after at least 48 h following hospitalization. ^2^ Symptoms are present at hospitalization or appear within 48 h in subjects recently hospitalized and/or administered an antibiotic therapy.

## Supplementary Materials

Supplementary Materials can be found at https://www.mdpi.com/2227-9059/8/9/335/s1. Supplementary Tables S1–S4: relative abundances of bacterial taxa in individual samples. Supplementary Figure S1: taxa displaying significantly different relative abundances in the following comparisons: C vs. CDI vs. rCDI1 (A); C vs. CDI vs. rCDI1 (B).
