# Supplementary material for: Microbiota in Clostridioides difficile-Associated Diarrhea: Comparison in Recurrent and Non-Recurrent Infections
Source: Biomedicines. 2020 Sep 8;8(9):335. doi: 10.3390/biomedicines8090335 (PMC7554755; doi:10.3390/biomedicines8090335)
Supplement: Supplementary file 1 [file biomedicines-08-00335-s001.zip › SUPPLEMENTARY Figure 1_kruskal wallis-ALTRI TAXA.pptx]

## Slide 1
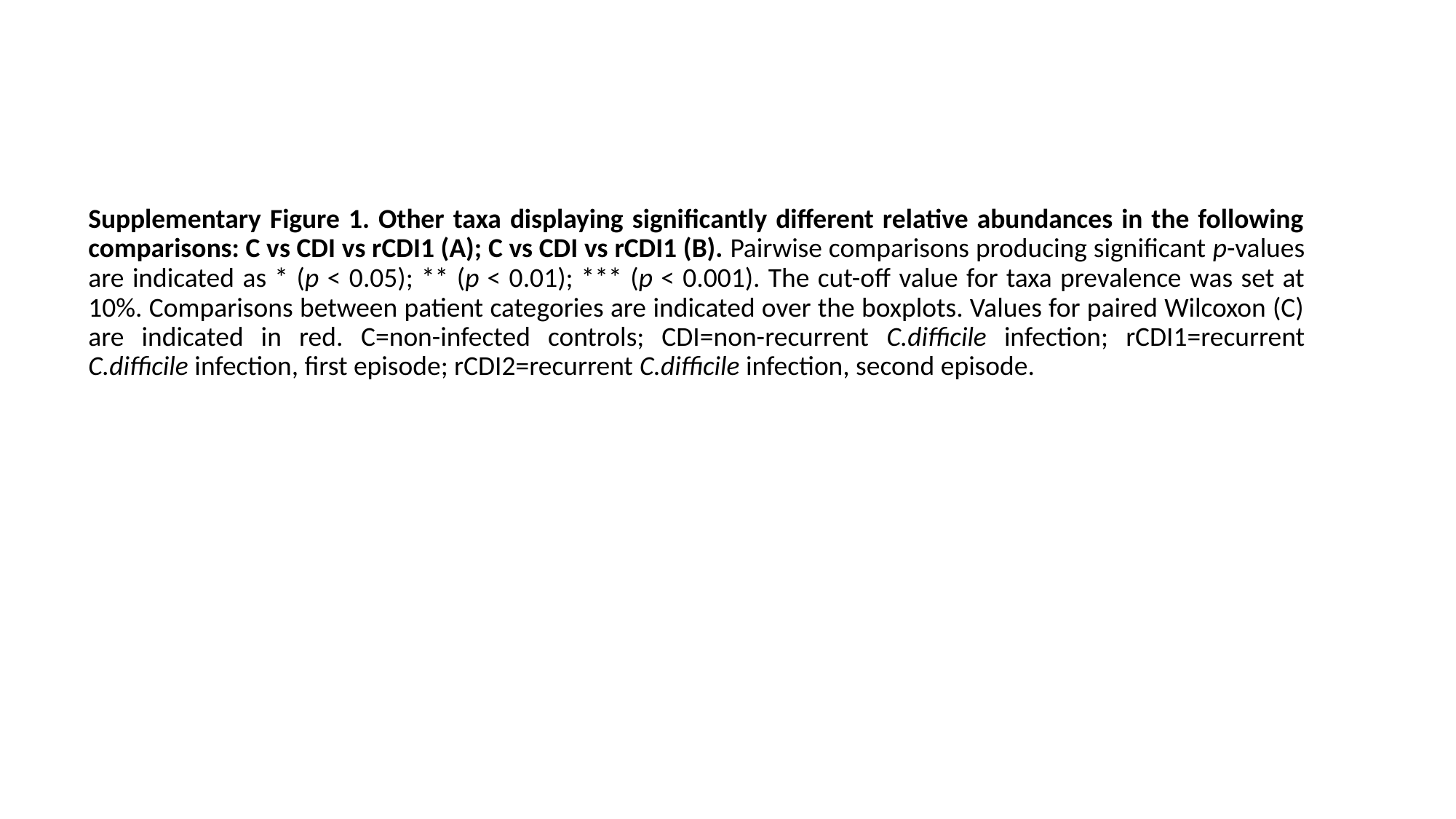

Supplementary Figure 1. Other taxa displaying significantly different relative abundances in the following comparisons: C vs CDI vs rCDI1 (A); C vs CDI vs rCDI1 (B). Pairwise comparisons producing significant p-values are indicated as * (p < 0.05); ** (p < 0.01); *** (p < 0.001). The cut-off value for taxa prevalence was set at 10%. Comparisons between patient categories are indicated over the boxplots. Values for paired Wilcoxon (C) are indicated in red. C=non-infected controls; CDI=non-recurrent C.difficile infection; rCDI1=recurrent C.difficile infection, first episode; rCDI2=recurrent C.difficile infection, second episode.

## Slide 2
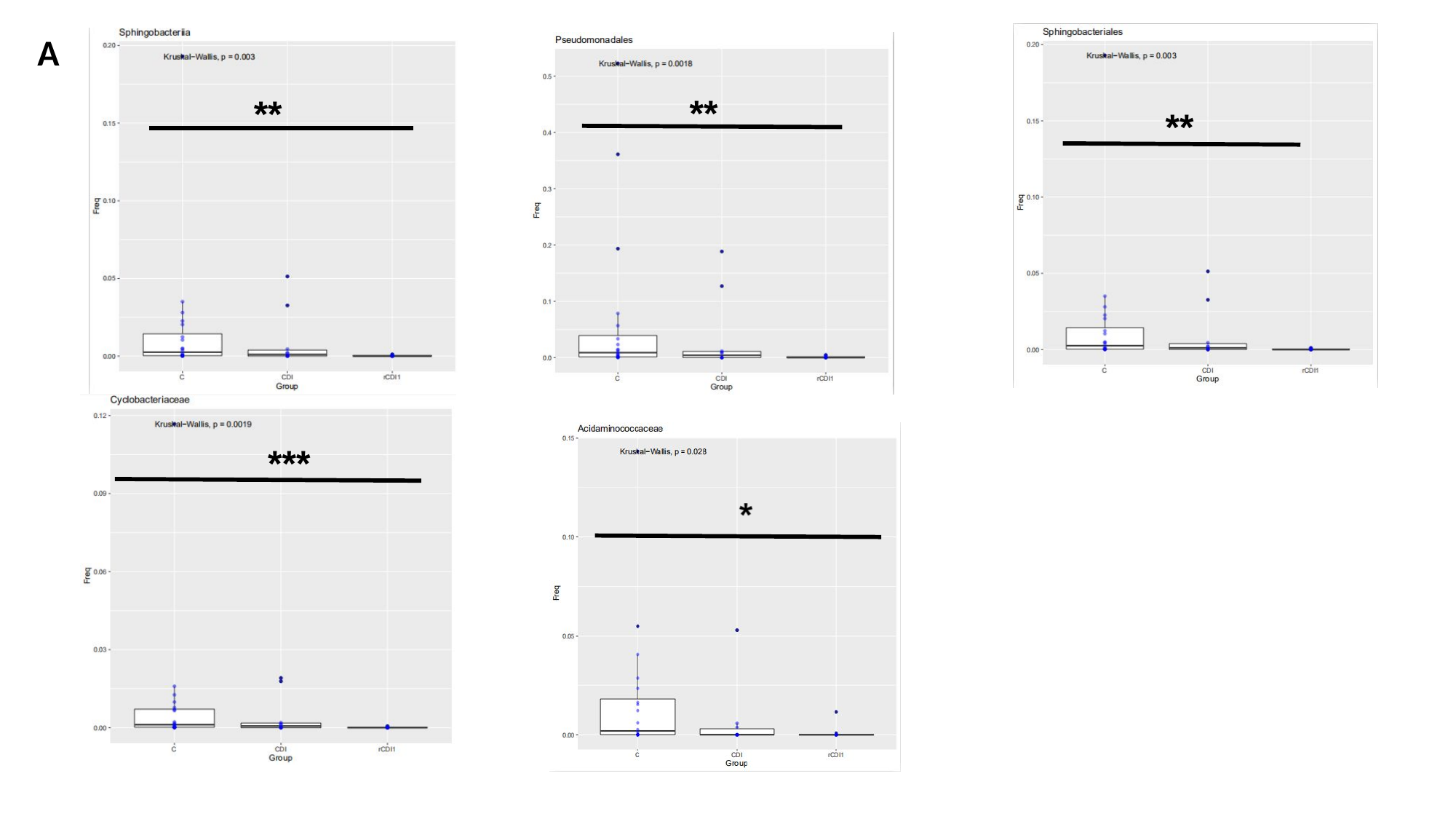

A
**
**
**
***

## Slide 3
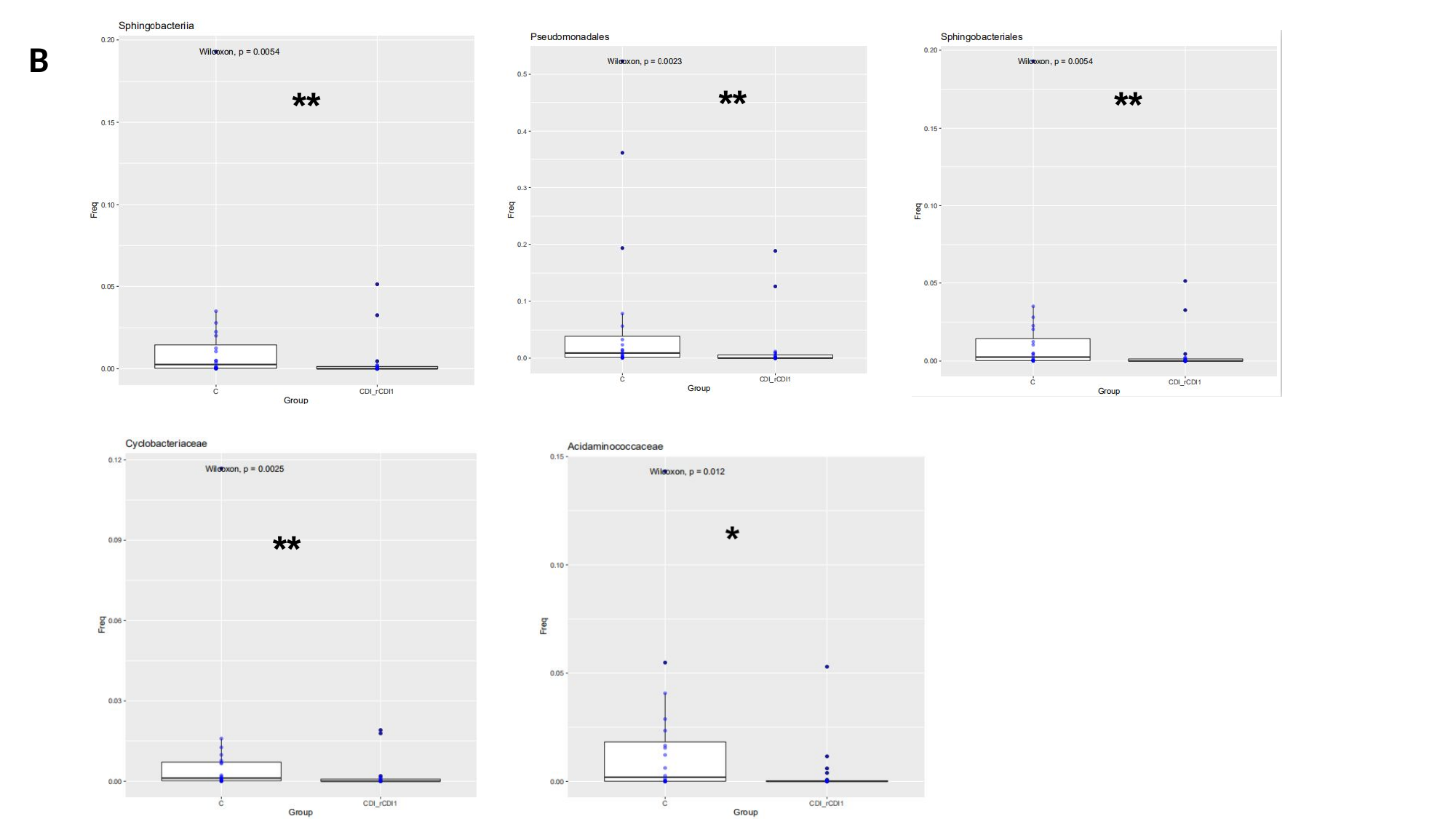

**
B
**
**
**

## Slide 4
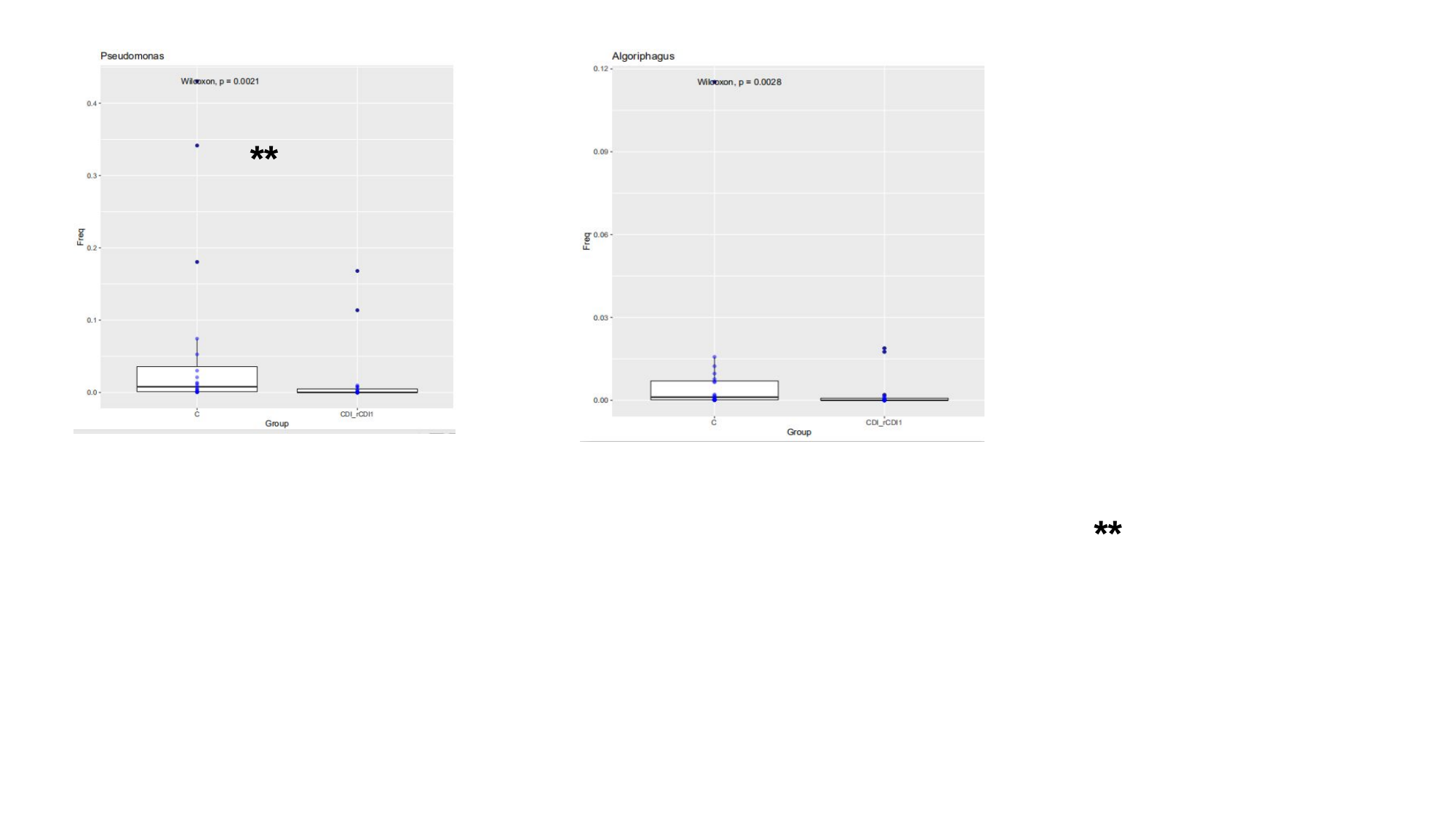

**
**
